# Supplementary material for: Diet composition and social environment determine food consumption, phenotype and fecundity in an omnivorous insect
Source: R Soc Open Sci. 2020 Apr 22;7(4):200100. doi: 10.1098/rsos.200100 (PMC7211883; doi:10.1098/rsos.200100)
Supplement: Titles and captions in a separate file [file rsos200100supp1.pdf]

Diet quality and social environment determine food consumption, phenotype and fecundity in an omnivorous insect

Yeisson Gutiérrez, Marion Fresch, David Ott, Jens Brockmeyer, Christoph Scherber

Quantification of lipid content

Insects were individually homogenized in methanol in a 1:30 ratio (w/v) with an Ultra-Turrax Tube Drive (IKA®-Werke GmbH & Co., Germany) at 6000 rpm during 3 min. An aliquot of 2 mL of each sample (avoiding suspended particles) was mixed with chloroform to reach a 2:1 chloroform:methanol solution and the vials were shaken in the cold for 1h. Recovery of the liquid phase was done after short centrifugation and 1.2 mL 150mM Ammonium bicarbonate was added to induce phase separation and force polar lipids into the organic phase. Samples were vigorously shaken for 10 min and finally centrifuged at 5000 rpm during 10 min at 4°C (Z326K, Hermle, Germany) and the lower organic phase was transferred to glass vials. The chloroform and methanol of the resulting organic phase (containing the lipids) were evaporated in a vacuum concentrator (RVC 2-18 CDplus, Martin Christ Gefriertrocknungsanlagen GmbH, Germany) and weighted using an analytical scale (Quintix 220 g x 0.1 mg, Sartorius, Germany).

Table S1. Diet recipes and diet constitution

Diet	EggPowder	Whey	Casein	Sugar	ingredients	Vanderzant	Methyl paraben	Agar	Water (ml)
3:1	9.128	24.017	25.914	10.941	70	2	0.3	4	300
1:1	9.128	16.366	17.654	26.845r	70	2	0.3	4	300

*all amounts expressed in g, except water.

Diet 3:1

	EggPowder	Whey	Casein	Sugar/Sucrose	Total	P:C	cal/g
**Carbs(g)**	0.14187	0.087805	0.094737	1	1.324412	3.001739	4.087045
**Prot(g)**	0.375539	1.8	1.8	0	3.975539		
**Fat(g)**	0.317122	0.164634	0.035526	0	0.517282		**fat/g**
**Cal**	4.923736	9.043902	8.431579	3.75	26.14922		0.08085
**total (g)**	0.834531	2.195122	2.368421	1	6.398074		
%	13.04348	34.3091	37.01772	15.62970245	100		

Diet 1:1

	EggPowder	Whey	Casein	Sugar/Sucrose	Total	P:C	cal/g
**Carbs(g)**	0.057825	0.02439	0.026316	1	1.108531	1.040175	4.069015
**Prot(g)**	0.153066	0.5	0.5	0	1.153066		
**Fat(g)**	0.129256	0.045732	0.009868	0	0.184856		**fat/g**
**Cal**	2.006871	2.512195	2.342105	3.75	10.61117		0.070886
**total (g)**	0.340148	0.609756	0.657895	1	2.607798		
%	13.04348	23.38203	25.22797	38.34652162	100		

Table S2. Models used for every response variable studied

Response variable	Model	function	Distribution	Random factor
Nymph survival	mixed-effects Cox models	coxme	NA	Experimental unit code
Development time	GLMM	glmer	Poisson	Experimental unit code
Individual body mass	GLMM	lme	Gaussian	Experimental unit code
Food consumption	GLM	glm	Gamma (inverse link)	NA
Cannibalism	GLM	glm	quasi-binomial (logit-link)	NA
Protein (total)	GLM	glm	Gamma (inverse link)	NA
Protein (Relative)	GLM	glm	quasi-binomial (logit-link)	NA
Lipids (total)	GLM	glm	Gamma (log-link)	NA
Lipids (relative)	GLM	glm	quasi-binomial (logit-link)	NA
Weekly egg production	GLMM	glmer	Negative binomial (1)	Experimental unit code
Total fecundity	GLM	glm	Negative binomial (1)	NA
Adult female lifespan	log rank test	survfit	NA	NA

Table S3. Summary for structural equation model for direct and indirect effects of the experimental treatments on several traits of female *A. domesticus.* SE = Standard errors.

Left-hand-side	Operator	Right-hand-side	Estimate	SE	z value	P value
**Regressions**						
Food consumption	~	Social environment	0.34	0.11	-3.03	**0.002**
Body mass	~	Social environment	-0.09	0.13	-0.66	0.508
Body mass	~	Diet composition	-0.05	0.13	-0.41	0.683
Body mass	~	Food consumption	0.48	0.11	4.52	**< 0.001**
Development time	~	Food consumption	-0.19	0.13	-1.54	0.124
Development time	~	Diet composition	0.40	0.13	3.17	**0.002**
**Covariance**						
Body mass	~~	Development time	0.28	0.13	2.18	**0.029**
**Variances**						
Food consumption			0.89	0.08	11.65	0.000
Body mass			0.73	0.11	6.52	0.000
Development time			0.81	0.11	7.66	0.000

Table S4. Summary for structural equation model for direct and indirect effects of the experimental treatments on several traits of male *A. domesticus.* SE = Standard errors.

Left-hand-side	Operator	Right-hand-side	Estimate	SE	z value	P value
**Regressions**						
Food consumption	~	Social environment	-0.08	0.13	-0.62	0.535
Body mass	~	Social environment	-0.11	0.11	-0.99	0.324
Body mass	~	Food consumption	0.27	0.11	2.41	**0.016**
Development time	~	Diet composition	0.23	0.12	2.03	**0.043**
**Covariance**						
Body mass	~~	Development time	0.24	0.12	2.08	**0.038**
**Variances**						
Food consumption			0.99	0.02	46.71	0
Body mass			0.91	0.06	14.29	0
Development time			0.95	0.05	17.60	0

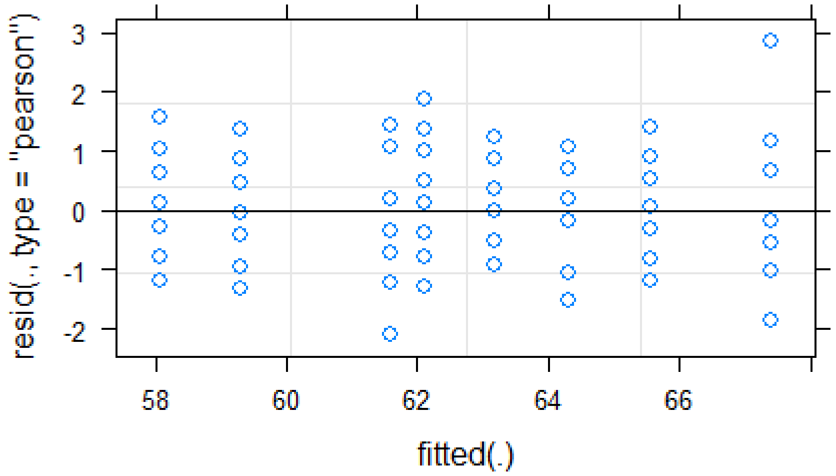

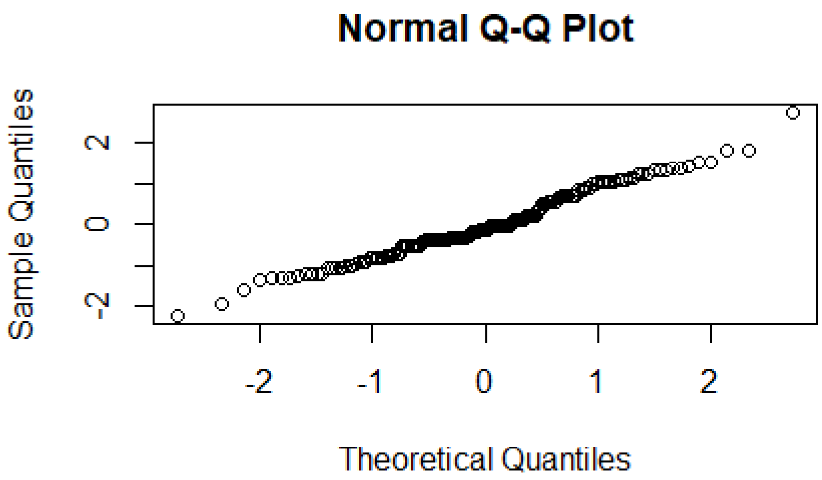

Fig. S1. Regression diagnostics for GLMM for development time of *Acheta domesticus*. Residual and quantile plots

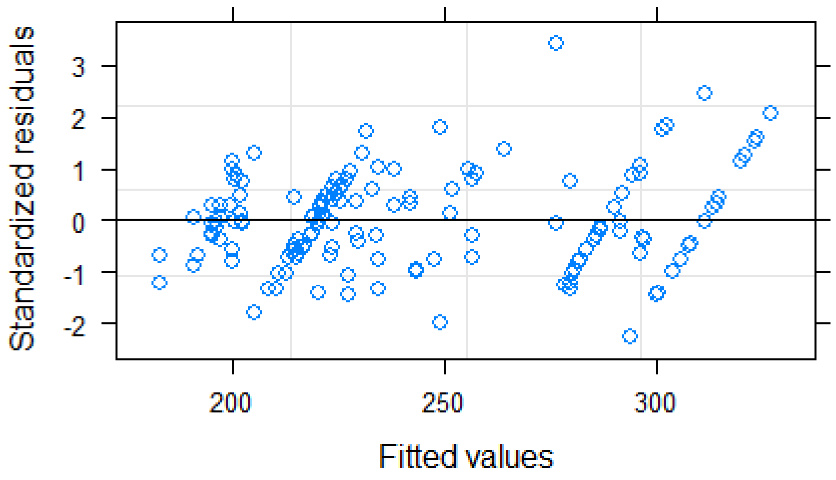

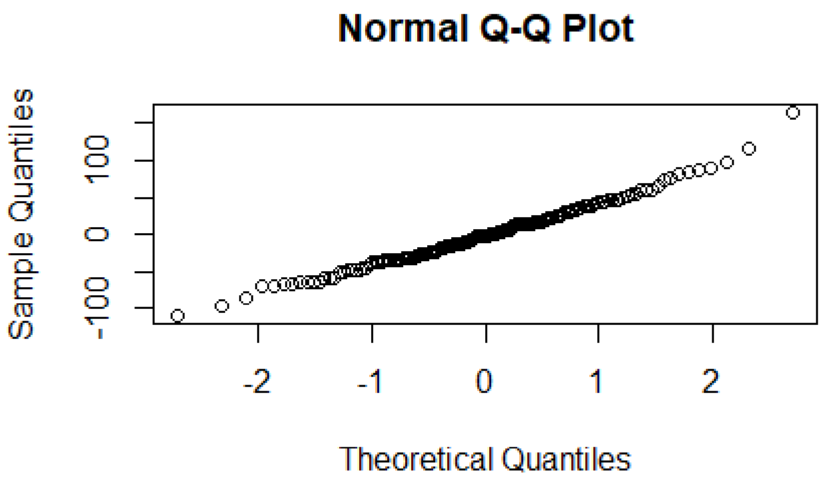

Fig. S2. Regression diagnostics for GLMM for body mass of *Acheta domesticus*. Residual and quantile plots

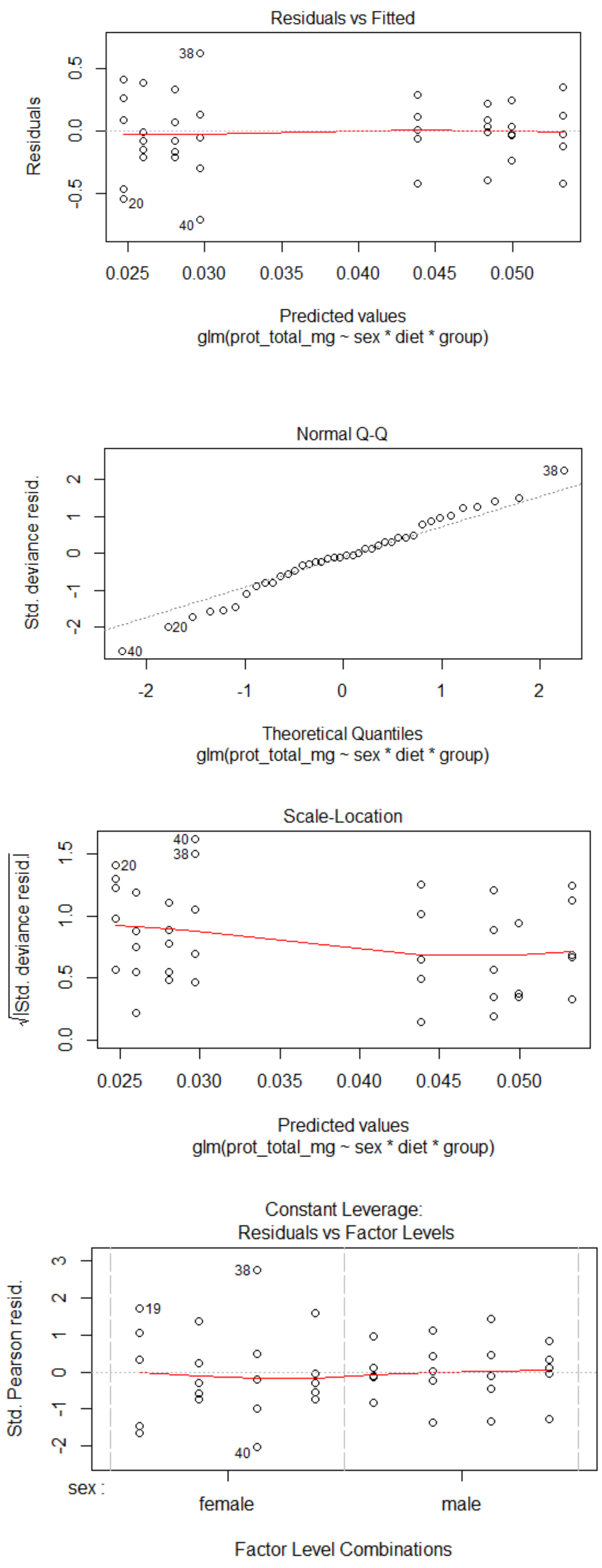

Fig. S3. Regression diagnostics for GLM for protein content of *Acheta domesticus*. Residual, quantile, scale-location and Cook's distance plots.

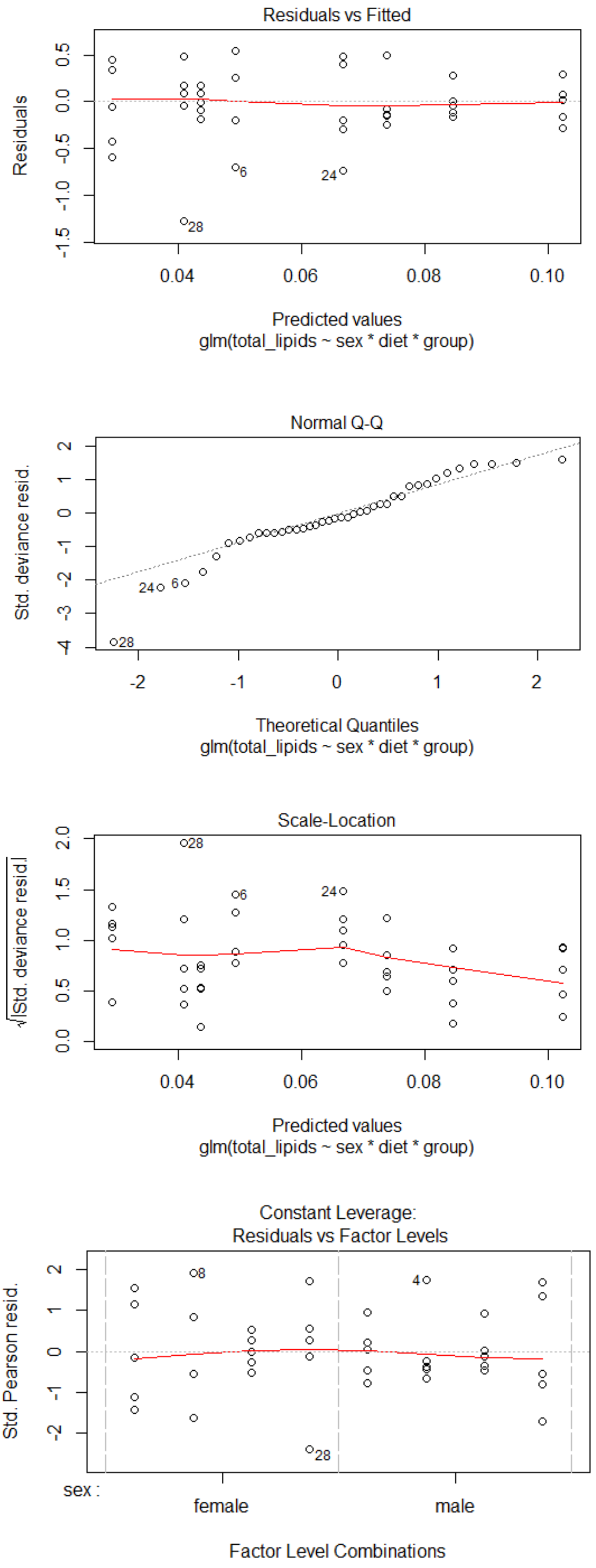

Fig. S4. Regression diagnostics for GLM for lipid content of *Acheta domesticus*. Residual, quantile, scale-location and Cook's distance plots.

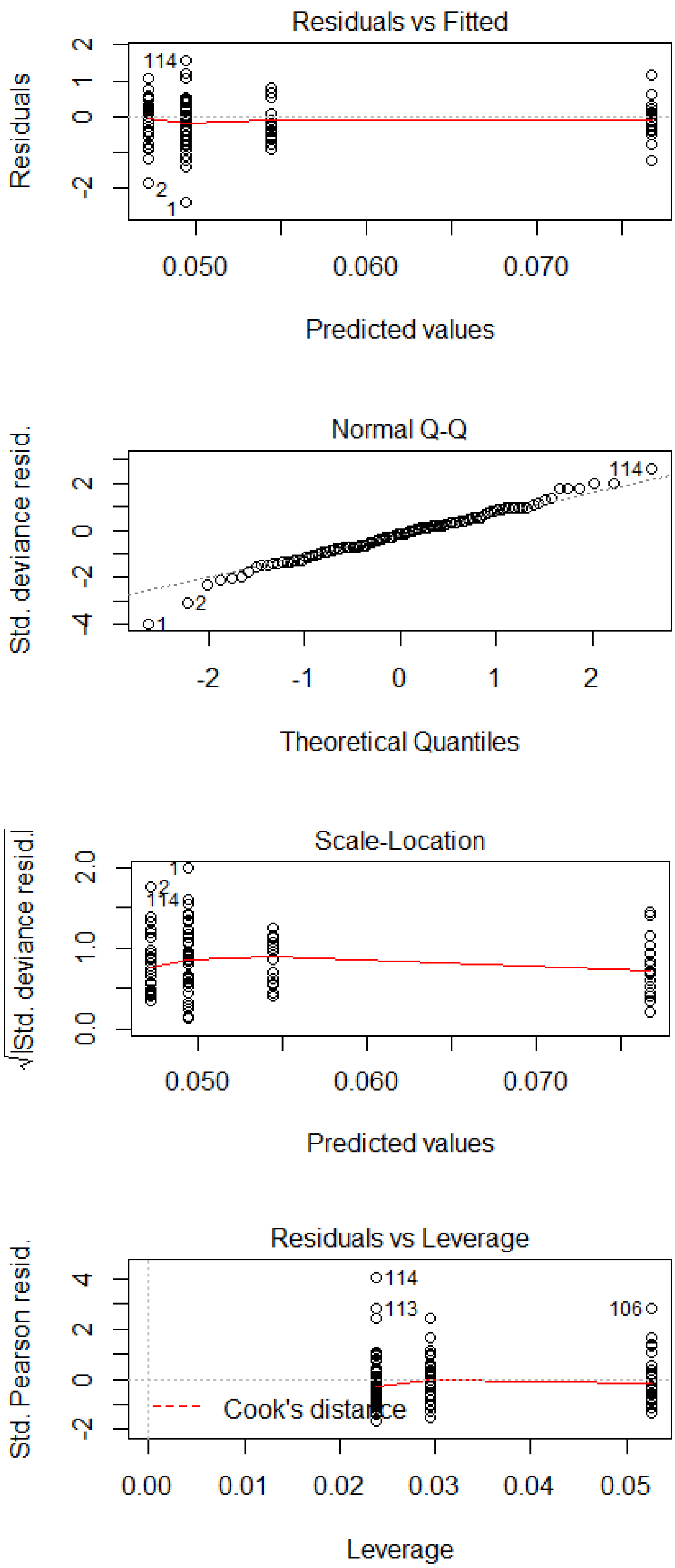

Fig. S5. Regression diagnostics for GLM for food consumption of solitary and grouped *Acheta domesticus.* Residual, quantile, scale-location and Cook's distance plots.

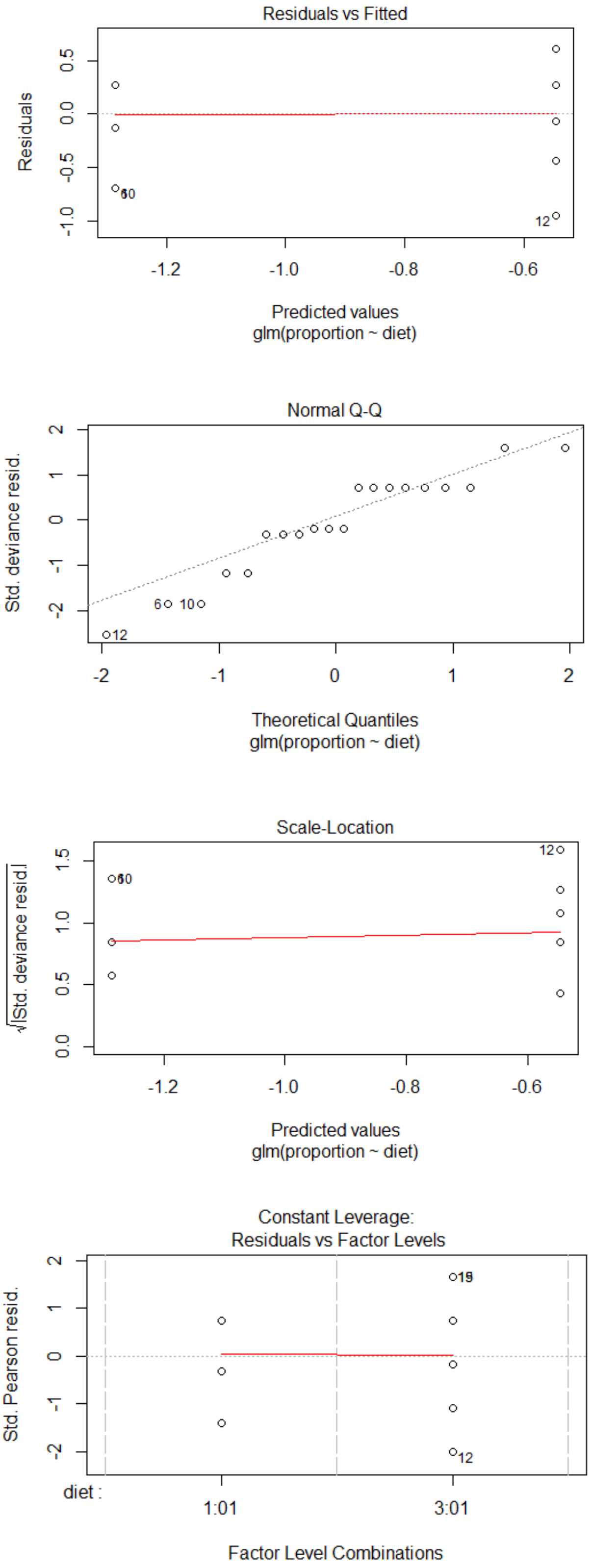

Fig. S6. Regression diagnostics for GLM for cannibalism of *Acheta domesticus*. Residual, quantile, scale-location and Cook's distance plots.

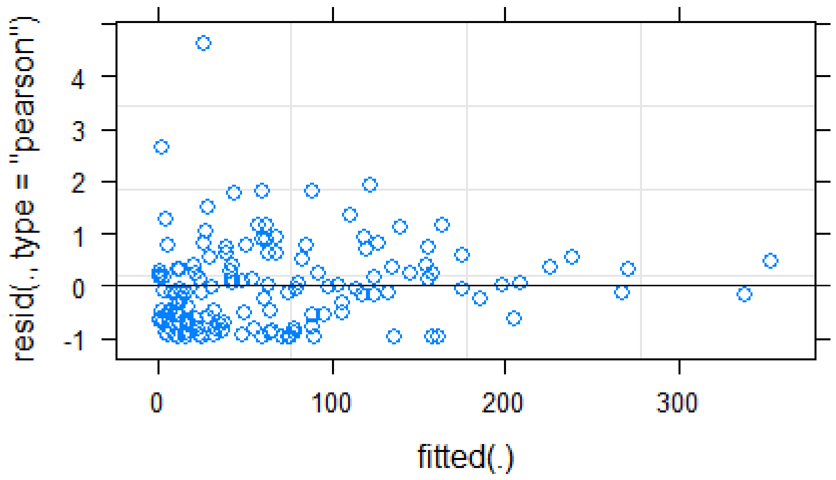

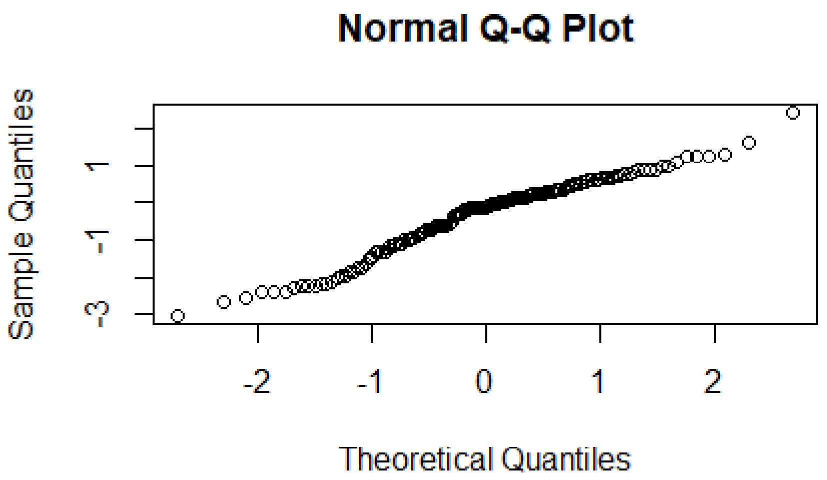

Fig. S7. Regression diagnostics for GLMM for weekly egg production of female *Acheta domesticus*. Residual and quantile plots

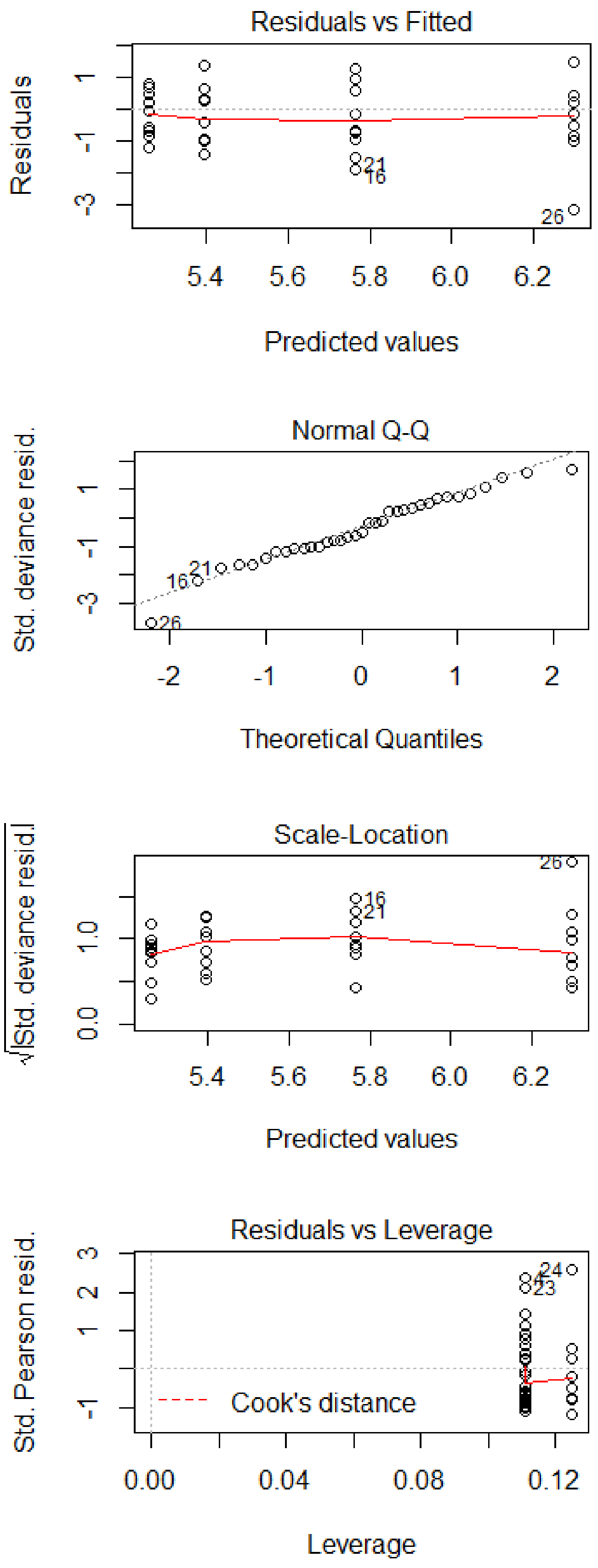

Fig. S8. Regression diagnostics for GLM for total egg production of female *Acheta domesticus*. Residual, quantile, scale-location and Cook's distance plots.
